# Base-Mediated One-Pot Synthesis of Aliphatic Diazirines for Photoaffinity Labeling

**DOI:** 10.3390/molecules22081389

**Published:** 2017-08-22

**Authors:** Lei Wang, Zetryana Puteri Tachrim, Natsumi Kurokawa, Fumina Ohashi, Yasuko Sakihama, Yasuyuki Hashidoko, Makoto Hashimoto

**Affiliations:** 1 Center for Drug Design, Academic Health Center, University of Minnesota, Minneapolis, MN 55455, USA; lei870610@gmail.com; 2 Division of Applied Bioscience, Graduate School of Agriculture, Hokkaido University, Kita 9, Nishi 9, Kita-ku, Sapporo 060-8589, Japan; z317_style@live.com (Z.P.T.); natsumi.k0420@gmail.com (N.K.); fumina28ohsei@gmail.com (F.O.); sakihama@abs.agr.hokudai.ac.jp (Y.S.); yasu-h@abs.agr.hokudai.ac.jp (Y.H.)

**Keywords:** photoaffinity labeling, one-pot synthesis, aliphatic diazirine, base-mediated reaction

## Abstract

Aliphatic diazirines have been widely used as prominent photophores for photoaffinity labeling owing to their relatively small size which can reduce the steric effect on the natural interaction between ligands and proteins. Based on our continuous efforts to develop efficient methods for the synthesis of aliphatic diazirines, we present here a comprehensive study about base-mediated one-pot synthesis of aliphatic diazirines. It was found that potassium hydroxide (KOH) can also promote the construction of aliphatic diazirine with good efficiency. Importantly, KOH is cheaper, highly available, and easily handled and stored compared with the previously used base, potassium *tert*-butoxide (*t*-BuOK). Gram-scale study showed that it owned great advantages in being used for the large-scale production of aliphatic diazirines. This protocol is highly neat and the desired products can be easily isolated and purified. As the first comprehensive study of the base-mediated one-pot synthesis of aliphatic diazirines, this work provided good insight into the preparation and utilization of diazirine-based photoaffinity labeling probes.

## 1. Introduction

**Scheme 1:**
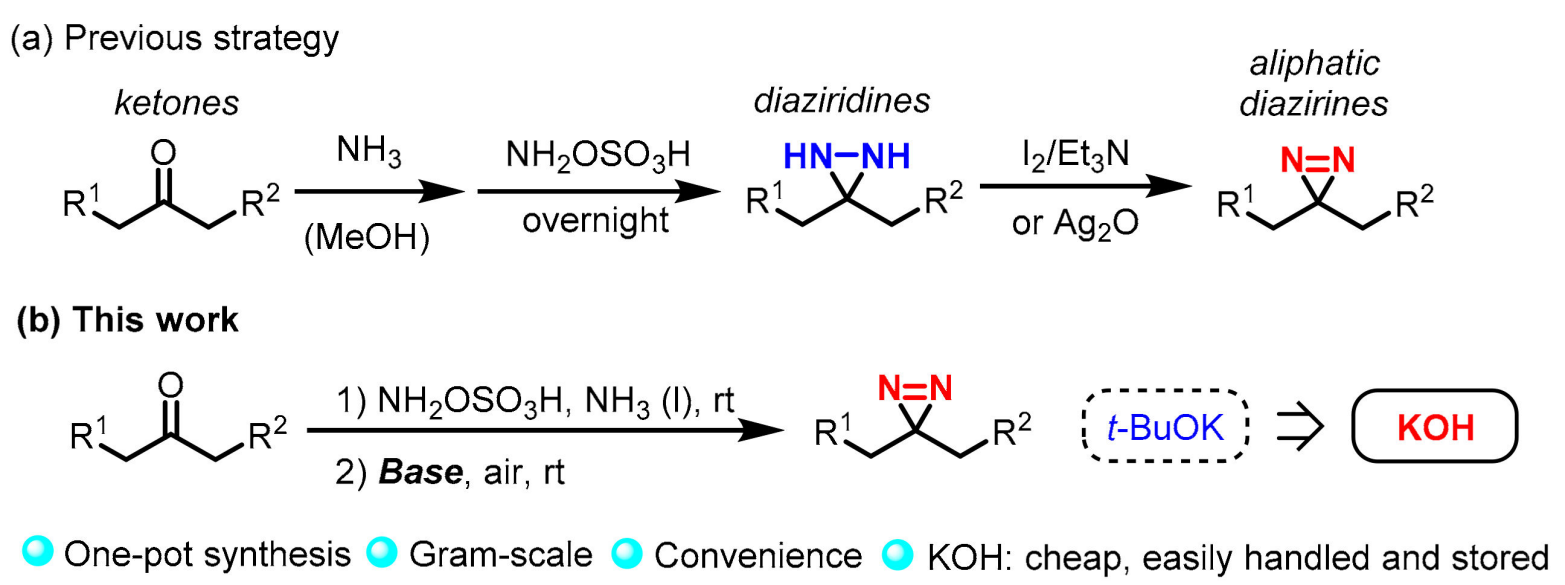
Previous (**a**) and base-mediated one-pot (**b**) synthesis of aliphatic diazirines.

Photoaffinity labeling [1,2,3,4,5] has emerged as a powerful methodology in the field of biology due to its availability for elucidating the interactions between bioactive molecules and proteins. Various photophores—including phenyl azides, benzophenones, and diazirines—are commonly employed in this field [6,7,8,9,10,11]. Under UV irradiation, photophores are converted into highly reactive intermediates which can form covalent bonds between ligands and proteins. The stable links in the components allow for further separation and detection. Among these photophores, aliphatic diazirines [12,13,14,15,16] have attracted tremendous attention due to their relatively small size, thus minimizing the steric effect of the natural interaction between ligands and proteins. For the conventional synthesis procedure of aliphatic diazirines [17,18], the ketones precursor was first treated with ammonia for several hours, then it was reacted with hydroxylamine-*O*-sulfonic acid (NH_2_OSO_3_H) to form diaziridines. After removal of the ammonia, diaziridines were further converted to diazirine in the presence of I_2_-TEA or Ag_2_O (Scheme 1a). Despite the wide applications, this protocol suffers many issues such as low yield and tedious procedures. To solve these issues, we have recently developed a novel one-pot synthesis of aliphatic diazirines by using *t*-BuOK under air [19]. In spite of its good efficiency, *t*-BuOK is a flammable base and it should be commonly handled and stored under inert gas. Furthermore, its relatively high price also limits the large-scale production of diazirines and the corresponding labeling probes. Herein, for the first time, we carried out a comprehensive study to screen the bases and other conditions, and it was found that KOH also could be used for one-pot synthesis of aliphatic diazirines with great efficiency (Scheme 1b). Significantly, it is cheaper, easily handled, and stored as well as being highly available in many labs. Gram-scale study confirmed its usability for the large-scale production of aliphatic diazirines. Furthermore, a kinetic study to elaborate the one-pot process was also performed on the basis of NMR analysis.

## 2. Results and Discussion

### 2.1. Reaction Optimization

We initiated our studies by using levulinic acid (**1a**) as a model substrate for reaction optimization, which was treated with 1.1 equivalent of NH_2_OSO_3_H in liquid ammonia at room temperature for 12 h to generate the corresponding diaziridine in situ. Then, base (3.3 equiv.) was gradually added into the mixture and the reaction was further stirred at room temperature under air for 2 h. Lithium amide was first tested and it was able to afford aliphatic diazirine (**2a**) at a 36% yield (Table 1, entry 1). Sodium amide was examined and a slightly higher yield of **2a** was obtained (44%, entry 2). Several metal hydrides were able to mediate the construction of diazirine (**2a**)**,** albeit with moderate yields (entries 3–5). Alkoxides were also utilized to promote the formation of products and the results indicated that a potassium base is preferred to sodium one (entries 6–8). Furthermore, NaOH and KOH [20,21,22,23,24,25] were employed and it was found that KOH was able to afford **2a** in 67% yield (entries 9 and 10). Although its efficiency was slightly lower compared to *t-*BuOK [19], KOH is easily handled, stored, and highly available in many labs. Its relatively low price makes it feasible for large-scale synthesis of aliphatic diazirines. Moreover, the by-products derived from OH anion can easily be removed in this reaction. Thus, we optimized KOH for the subsequent study. The reaction carried out at either low temperature (entry 11) or in methanolic ammonia (entry 12) caused a low yield of product (**2a**), indicating that both temperature and concentration of ammonia are crucial for the generation of diazirine in this protocol.

### 2.2. Scope Investigation

**Scheme 2:**
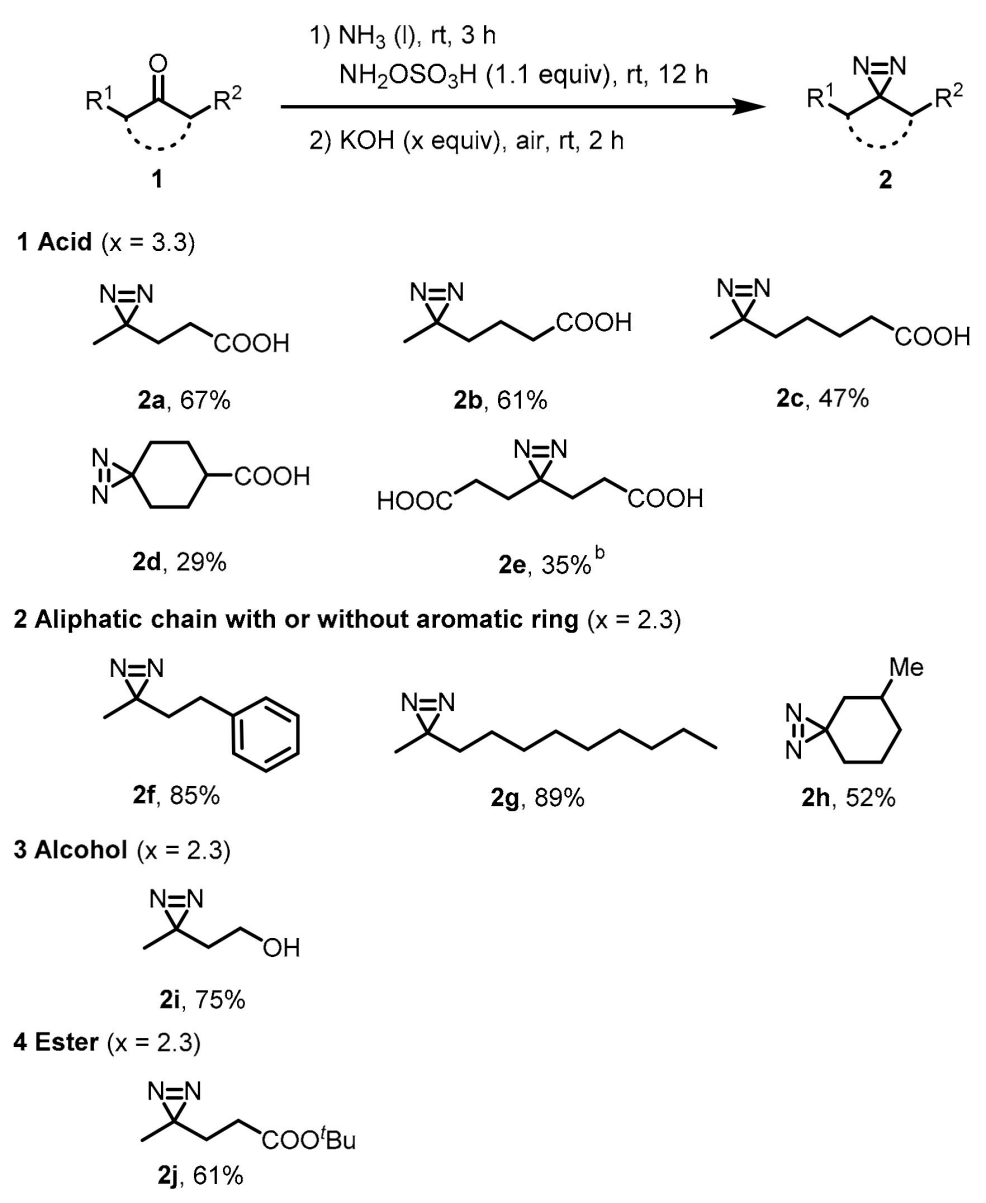
Scope investigation of one-pot synthesis of aliphatic diazirine with KOH ^a^. ^a^ Unless otherwise mentioned, **1** (2 mmol) and NH_2_OSO_3_H (1.1 equiv.) was added into liquid ammonia (8 mL) at −78 °C and the reaction mixture was stirred at room temperature for 12 h until base was added. After the addition of base, the reaction was further stirred at room temperature under air for 2 h. ^b^ 4.3 equivalent of KOH was used.

With the optimized condition in hand, the generality of the protocol was evaluated with different ketone procedures. A wide range of substituted ketones—including acid, alcohol, ester, and hexatomic rings—were tolerated under this condition to be converted into the corresponding diazirine products. When monoacid substrates were tested, 3.3 equivalents of KOH were required to create the desired product and the corresponding diazirines were obtained in moderate to good yield (Scheme 2, **2a**–**d**). A binary acid derivative (**1e**) was subjected to the optimal reaction with 4.3 equivalents of KOH, and the desired product (**2e**) was isolated in 35% yield. Neutral ketones with aliphatic chain or aromatic ring are good substrates (**2f**–**h**) and up to an 89% yield was given for compound (**2g**). 3-Azibutanol (**2i**), a widely utilized photophore, was readily synthesized with a 75% yield. Compared to the previously synthetic method for **2i**, the KOH-mediated procedure showed a relatively good efficiency. Furthermore, *t*-butyl alcohol ester was also tolerated in the optimal condition, and the desired product was isolated in 61% yield (**2j**). The spectroscopic data were summarized in Supplementary Materials.

### 2.3. Kinetic Study of One-Pot Synthesis of Aliphatic Diaizirine

**Scheme 3:**
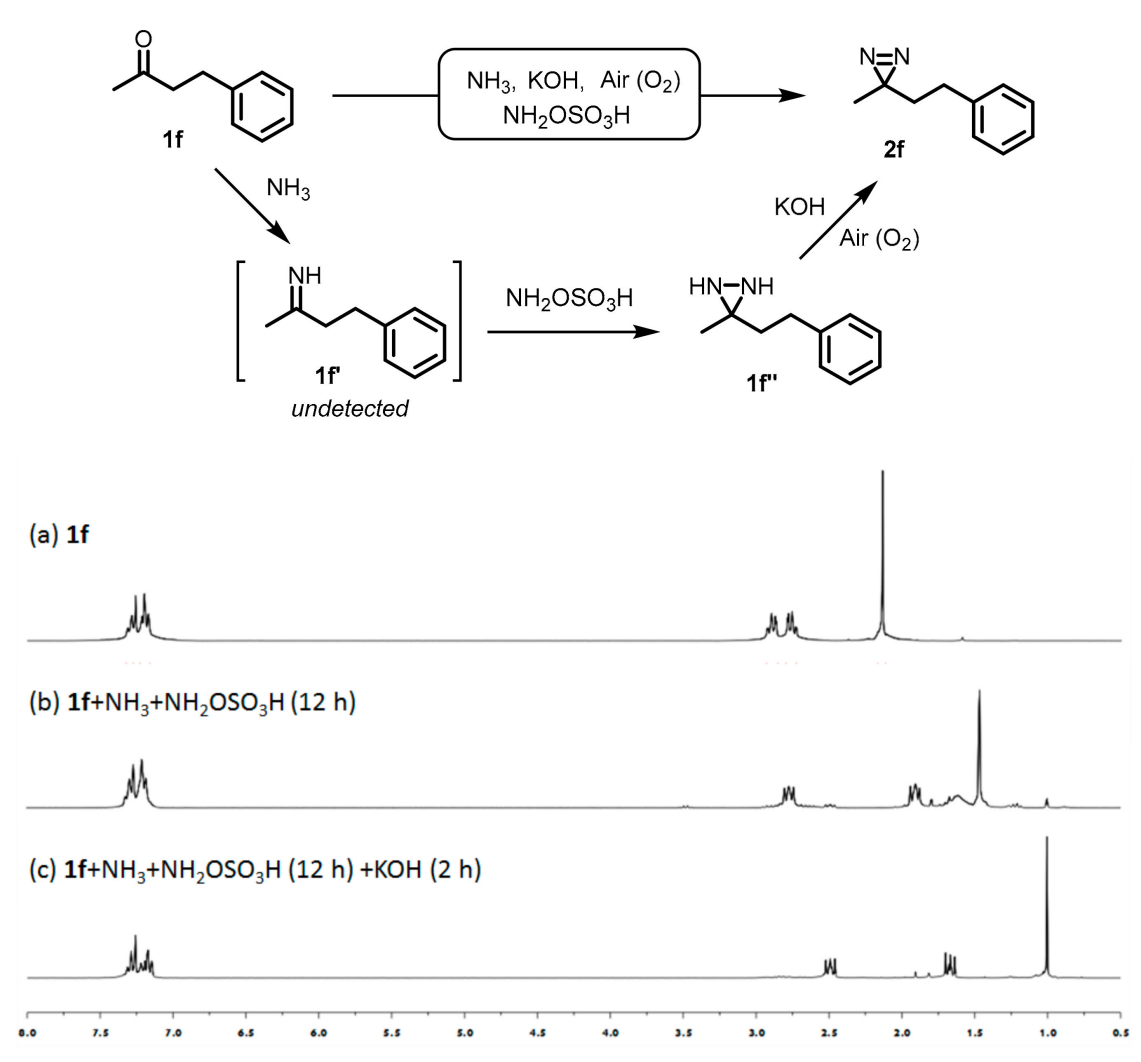
One-pot synthesis of aliphatic diazirine **2f**. The reaction stages were monitored using ^1^H-NMR with CDCl_3._ (**a**) starting material **1f**; (**b**) **1f** was treated with NH_2_OSO_3_H in liquid ammonia for 12 h; (**c**) The residue was further treated with KOH in liquid ammonia under air for 2 h.

To investigate the one-pot process, we performed a kinetic study based on ^1^H-NMR analysis. Aliphatic ketone (**1f**) (Scheme 3a) was initially treated with liquid ammonia at room temperature, while no imine (**1f′**) was observed although the treatment was continued to 12 h. When **1f** was subjected to the reaction with NH_2_OSO_3_H in liquid ammonia at room temperature for 12 h, the corresponding diaziridine (**1f′′**) was detected (Scheme 3b). To be noted, a trace amount of diazirine (**2f**) was generated at this stage which can be detected by ^1^H-NMR spectrum. Following with further treatment by KOH for 2 h, the formation of diazirine (**2f**) was dramatically improved (Scheme 3c). Furthermore, no other by-products were distinctly detected in the spectrum during the formation of diaziridine as well as diazirine. These results indicated that diaziridine as the precursor can be directly converted to diazirine in KOH-mediated one-pot reaction. This method is useful with the isolation and purification of the final products being more convenient.

### 2.4. Gram-Scale Synthesis

**Scheme 4:**
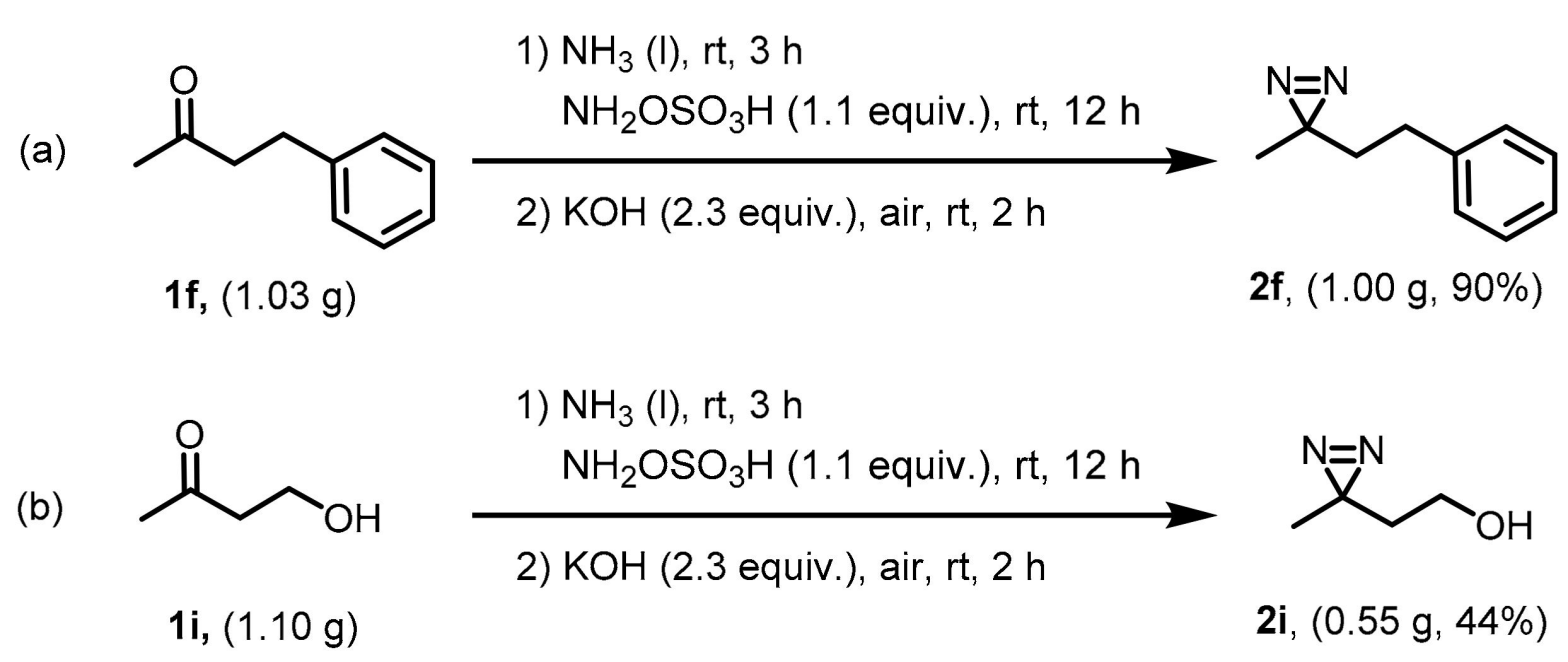
Gram-scale synthesis of **2f** (**a**) and **2i** (**b**).

To explore the behavior of this protocol for large-scale synthesis of aliphatic diazirines, we designed gram-scale reactions. Compound **1f** (1.03 g) was subjected to the optimized conditions and the desired product (**2f**) can be readily isolated at a 90% yield (Scheme 4a). It undoubtedly confirmed the advantages and applicability of KOH-mediated synthetic strategy for the large-scale synthesis of aliphatic diazirines. 3-Azibutanol (**2i**) is a widely used photophore that labels active molecules in the field of photoaffinity labeling [26,27,28,29,30]. While many syntheses suffered relatively low yields. By using the KOH-mediated one-pot strategy, we can synthesize **2i** in a 75% yield (Scheme 2). It is a great improvement compared with previous synthetic methods as well as the *t*-BuOK-mediated one. A gram-scale synthesis was carried out and the protocol was able to afford **2i** at a 44% yield from gram-scale **1i** (Scheme 4b), thus indicating the good applicability of KOH-mediated one-pot strategy in large-scale synthesis.

## 3. Materials and Methods

### 3.1. General Procedures

All reactions were carried out in a metal sealed tube (300 mL, 10 MPa, 200 °C). The NMR spectra were measured on JEOL EX-270 (JEOL, Tokyo, Japan). All solvents were of reagent grade and purified using the appropriate methods. HRMS spectra were obtained with a Waters UPLC ESI-TOF mass spectrometer (Waters, Milford, CT, USA).

### 3.2. Typical Procedures for One-Pot Synthesis of Aliphatic Diazirines from Corresponding Ketones

#### 3.2.1. Acidic Substrates (**1a**–**e**)

Ketone (2 mmol) was dissolved in liquid NH_3_ (8 mL) in a sealed tube at −78 °C. Hydroxylamine-*O*-sulfonic acid (1.1 equiv.) was carefully added at the same temperature. After closing the tube, the reaction mixture was stirred at room temperature for 12 h, before it was cooled at −78 °C and subsequently opened. KOH (3.3 equiv. for **1a**–**d** and 4.3 equiv. for **1e**) was added to liquid NH_3_ mixture and then the tube was sealed. The reaction mixture was further stirred at room temperature under air for 2 h. The ammonia was gradually removed at room temperature in a fume hood. The residue was dissolved with water (30 mL) which was adjust pH to 2 with HCl aq, then extracted twice with Et_2_O (30 mL). The organic layer was washed with brine, dried over MgSO_4_, and evaporated. The residue was subjected to silica-gel column chromatography to afford the diazirinyl products.

#### 3.2.2. Neutral Substrates (**1f**–**j**)

Ketone (2 mmol) was dissolved in liquid NH_3_ (8 mL) in a sealed tube at −78 °C. Hydroxylamine-*O*-sulfonic acid (1.1 equiv.) was carefully added at same temperature. After closing the tube, the reaction mixture was stirred at room temperature for 12 h, before it was cooled at −78 °C and subsequently opened. KOH (2.3 equiv.) was added to liquid NH_3_ mixture then the tube was sealed. The reaction mixture was stirred at room temperature under air for 2 h. The ammonia was gradually removed at room temperature under a fume hood. The residue was partitioned between water (30 mL) and Et_2_O (30 mL). The aqueous layer was extracted twice with Et_2_O (30 mL). The combined organic layer was washed by brine, dried over MgSO_4_, and evaporated. The residue was subjected to silica-gel column chromatography to afford the diazirinyl products.

*3-(3-Methyl-3H-diazirin-3-yl)propanoic acid* (**2a**): Pale yellow oil, ^1^H-NMR (270 MHz, CDCl_3_) δ ppm: 11.88 (s, 1H), 2.26 (t, *J* = 7.6 Hz, 2H), 1.73 (t, *J* = 7.6 Hz, 2H), 1.05 (s, 3H); ^13^C-NMR (68 MHz, CDCl_3_) 178.3, 29.1, 28.2, 24.8, 19.2; HRMS (ESI) *m*/*z* [M + Na]^+^ calcd. for C_5_H_8_N_2_O_2_Na 151.0483, found 151.0508.

*4-(3-Methyl-3H-diazirin-3-yl)butanoic acid* (**2b**): Pale yellow oil, ^1^H-NMR (270 MHz, CDCl_3_) δ ppm: 2.35 (t, *J* = 7.2 Hz, 2H), 1.48–1.57 (m, 2H), 1.38–1.44 (m, 2H), 1.02 (s, 3H); ^13^C-NMR (68 MHz, CDCl_3_) 179.7, 33.4, 33.1, 25.2, 19.5, 19.0; HRMS (ESI) *m*/*z* [M + Na]^+^ calcd. for C_6_H_10_N_2_O_2_Na 165.0640, found 165.0641.

*5-(3-Methyl-3H-diazirin-3-yl)pentanoic acid* (**2c**): Pale yellow oil, ^1^H-NMR (270 MHz, CDCl_3_) δ ppm: 2.34 (t, *J* = 7.4 Hz, 2H), 1.56–1.67 (m, 2H), 1.35–1.41 (m, 2H), 1.19–1.29 (m, 2H), 1.01 (s, 3H); ^13^C-NMR (68 MHz, CDCl_3_) 179.9, 33.9, 33.7, 25.5, 24.0, 23.4, 19.6; HRMS (ESI) *m*/*z* [M + Na]^+^ calcd. for C_7_H_12_N_2_O_2_Na 179.0796, found 179.0778.

*1,2-Diazaspiro[2.5]oct-1-ene-6-carboxylic acid* (**2d**): Pale yellow solid, ^1^H-NMR (270 MHz, CDCl_3_) δ ppm: 2.47–2.57 (m, 1H), 2.02–2.11 (m, 2H), 1.82–1.92 (m, 2H), 1.61–1.73 (m, 2H), 0.87 (dt, *J* = 14.1, 3.6 Hz, 2H); ^13^C-NMR (68 MHz, CDCl_3_) 181.6, 41.2, 29.8, 27.3, 26.4; HRMS (ESI) *m*/*z* [M + H]^+^ calcd. for C_7_H_11_N_2_O_2_ 155.0821, found 155.0809.

*3,3′-(3H-Diazirine-3,3-diyl)dipropanoic acid* (**2e**): Pale yellow solid, ^1^H-NMR (270 MHz, CDCl_3_) δ ppm: 2.11 (t, *J* = 7.5 Hz, 4H), 1.72 (t, *J* = 7.5 Hz, 4H); ^13^C-NMR (68 MHz, CDCl_3_) 176.2, 29.1, 28.9, 28.7; HRMS (ESI) *m*/*z* [M + Na]^+^ calcd. for C_7_H_10_N_2_O_4_Na 209.0538, found 209.0551.

*3-Methyl-3-phenethyldiaziridine* (**1f′′**): Pale yellow oil, ^1^H-NMR (270 MHz, CDCl_3_) δ ppm: 7.27–7.32 (m, 2H), 7.18–7.22 (m, 3H), 2.77 (t, *J* = 7.8 Hz, 2H), 1.91 (t, *J* = 7.8 Hz, 2H), 1.61–1.70 (m, 2H), 1.47 (s, 3H); ^13^C-NMR (68 MHz, CDCl_3_) δ 141.1, 128.5, 128.2, 126.1, 55.0, 40.4, 31.3, 22.9; HRMS (ESI) *m*/*z* [M + H]^+^ calcd. for C_10_H_15_N_2_ 163.1235, found 163.1238.

*3-Methyl-3-phenethyl-3H-diazirine* (**2f**): Pale yellow oil, ^1^H-NMR (270 MHz, CDCl_3_) δ ppm: 7.26–7.31 (m, 2H), 7.15–7.22 (m, 3H), 2.46–2.52 (m, 2H), 1.64–1.70 (m, 2H), 1.00 (s, 3H); ^13^C-NMR (68 MHz, CDCl_3_) 140.9, 128.6, 128.3, 126.2, 36.3, 30.0, 25.6, 19.8; HRMS (ESI) *m*/*z* [M + H]^+^ calcd. for C_10_H_13_N_2_ 161.1079, found 161.1085.

*3-Methyl-3-nonyl-3H-diazirine* (**2g**): Pale yellow oil, ^1^H-NMR (270 MHz, CDCl_3_) δ ppm: 1.25–1.35 (m, 16H), 0.99 (s, 3H), 0.88 (t, *J* = 6.2 Hz, 3H); ^13^C-NMR (68 MHz, CDCl_3_) 34.2, 31.8, 29.33, 29.31, 29.2, 29.1, 23.9, 22.5, 19.8, 13.9; HRMS (ESI) *m*/*z* [M + H]^+^ calcd. for C_11_H_23_N_2_ 183.1861, found 183.1888.

*5-Methyl-1,2-diazaspiro[2.5]oct-1-ene* (**2h**): Pale yellow oil, ^1^H-NMR (270 MHz, CDCl_3_) δ ppm: 1.58–1.81 (m, 5H), 1.36–1.45 (m, 1H), 1.00–1.10 (m, 1H), 0.91 (d, *J* = 6.4 Hz, 3H), 0.50–0.59 (m, 2H); ^13^C-NMR (68 MHz, CDCl_3_) 39.8, 33.6, 31.0, 30.9, 28.3, 23.5, 21.8; HRMS (ESI) *m*/*z* [M + H]^+^ calcd. for C_7_H_13_N_2_ 125.1079, found 125.1084.

*2-(3-Methyl-3H-diazirin-3-yl)ethanol* (**2i**): Pale yellow oil, ^1^H-NMR (270 MHz, CDCl_3_) δ ppm: 3.54 (t, *J* = 6.3 Hz, 2H), 1.65 (t, *J* = 6.3 Hz, 2H), 1.08 (s, 3H); ^13^C-NMR (68 MHz, CDCl_3_) 57.6, 36.9, 24.1, 20.1; HRMS (ESI) *m*/*z* [M + H]^+^ calcd. for C_4_H_9_N_2_O 101.0715, found 101.0738.

*Ttert-butyl 3-(3-methyl-3H-diazirin-3-yl)propanoat*e (**2j**): ^1^H-NMR (270 MHz, CDCl_3_) δ ppm: 2.03 (t, *J* = 7.7 Hz, 2H), 1.59 (t, *J* = 7.7 Hz, 2H), 1.38 (s, 9H), 0.95 (s, 3H); ^13^C-NMR (68 MHz, CDCl_3_) 171.7, 80.6, 30.0, 29.6, 27.9, 25.1, 19.5; HRMS (ESI) *m*/*z* [M + H]^+^ calcd. for C_9_H_17_N_2_O_2_ 185.1285, found 185.1302.

## 4. Conclusions

In conclusion, we reported a comprehensive study about the base-mediated one-pot synthesis of aliphatic diazirines. KOH, a cheap, easily-handled and stored base, was optimized for the one-pot reaction. Many precious diazirines were prepared in moderate to excellent yields. Kinetic study showed that diaziridine as the intermediate can be directly converted to diazirine after the addition of KOH under air. Gram-scale synthesis indicated its good applicability in the large-scale synthesis of aliphatic diazirines for photoaffinity labeling. The reactions are pretty neat and products can be easily isolated and purified. This protocol will be helpful for effective synthesis of diazirine-based photoaffinity label ligands to elucidate functional analysis of biological activities.

## Figures and Tables

**Table 1:**
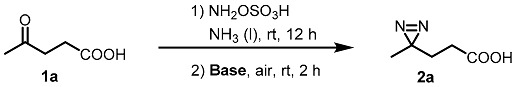
Reaction optimization of one-pot synthesis for aliphatic diazirine **2a** ^a^.

Entry	Solvent	Base	Temp	Yield (%) ^b^
1	NH_3_	LiNH_2_	RT	36
2	NH_3_	NaNH_2_	RT	44
3	NH_3_	NaH	RT	47
4	NH_3_	MgH_2_	RT	20
5	NH_3_	CaH_2_	RT	17
6	NH_3_	EtONa	RT	45
7	NH_3_	MeONa	RT	45
8	NH_3_	MeOK	RT	63
9	NH_3_	NaOH	RT	28
10	NH_3_	KOH	RT	67
11	NH_3_	KOH	0 °C	11
12	NH_3_/MeOH ^c^	KOH	RT	5

^a^ Unless otherwise mentioned, **1a** (2 mmol) and NH_2_OSO_3_H (1.1 equiv.) was added to liquid ammonia (8 mL) at −78 °C and the reaction mixture was stirred at room temperature for 12 h until base (3.3 equiv.) was added. After the addition of base, the reaction was further stirred at room temperature under air for 2 h. ^b^ Isolated yields were presented. ^c^ Ammonia gas was bubbled into MeOH (8 mL) at 0 °C for 30 min.

## Supplementary Materials

Copies of the ^1^H- and ^13^C-NMR spectra are available online.
