# Supplementary material for: Base-Mediated One-Pot Synthesis of Aliphatic Diazirines for Photoaffinity Labeling
Source: Molecules. 2017 Aug 22;22(8):1389. doi: 10.3390/molecules22081389 (PMC6152361; doi:10.3390/molecules22081389)
Supplement: Supplementary file 1 [file molecules-22-01389-s001.pdf]

Supporting Information

Base-Mediated One-Pot Synthesis of Aliphatic Diazirines for Photoaffinity Labeling

Lei Wang [†], Zetryana Puteri Tachrim, Natsumi Kurokawa, Fumina Ohashi, Yasuko Sakihama, Yasuyuki Hashidoko and Makoto Hashimoto [*]

Division of Applied Bioscience, Graduate School of Agriculture, Hokkaido University, Kita 9, Nishi 9, Kita-ku, Sapporo 060-8589, Japan.

[†]Present address: Center for Drug Design, Academic Health Center, University of Minnesota, Minneapolis, Minnesota 55455, United States.

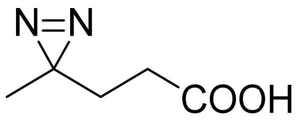
N=N
COOH

2a

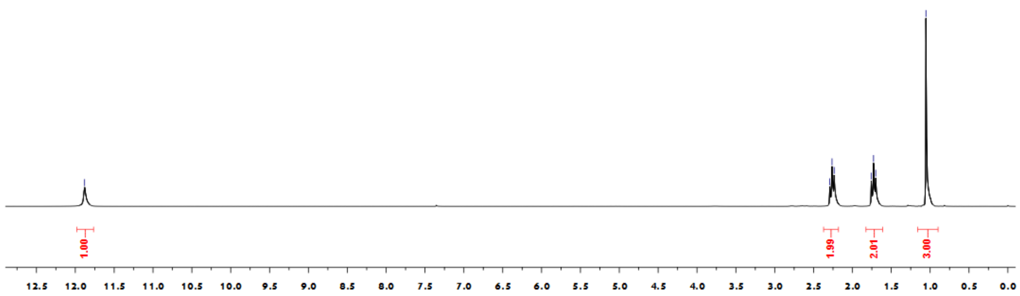
11.88
2.29
2.26
2.23
1.76
1.73
1.70
1.05
1.00
1.99
2.01
3.00

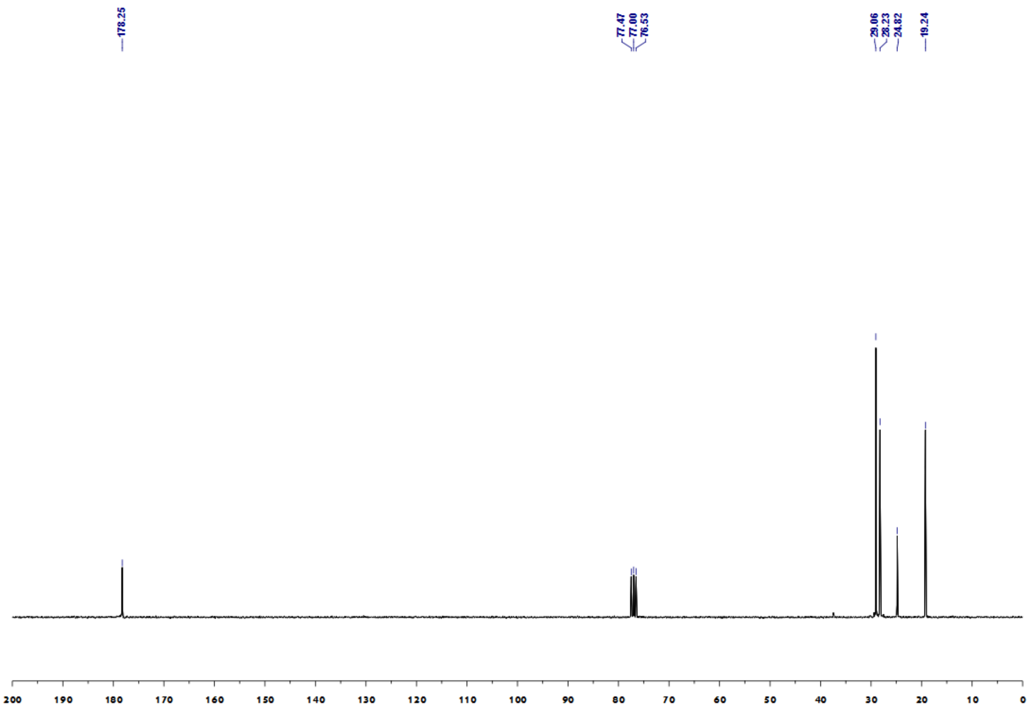
178.25
77.47
77.00
76.53
29.06
28.23
24.82
19.24

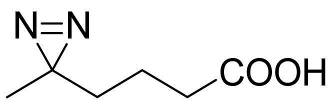

2b

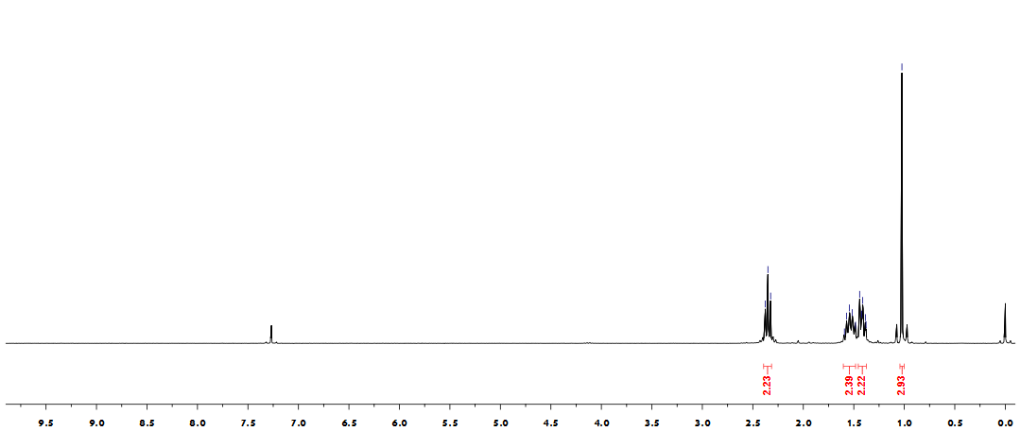

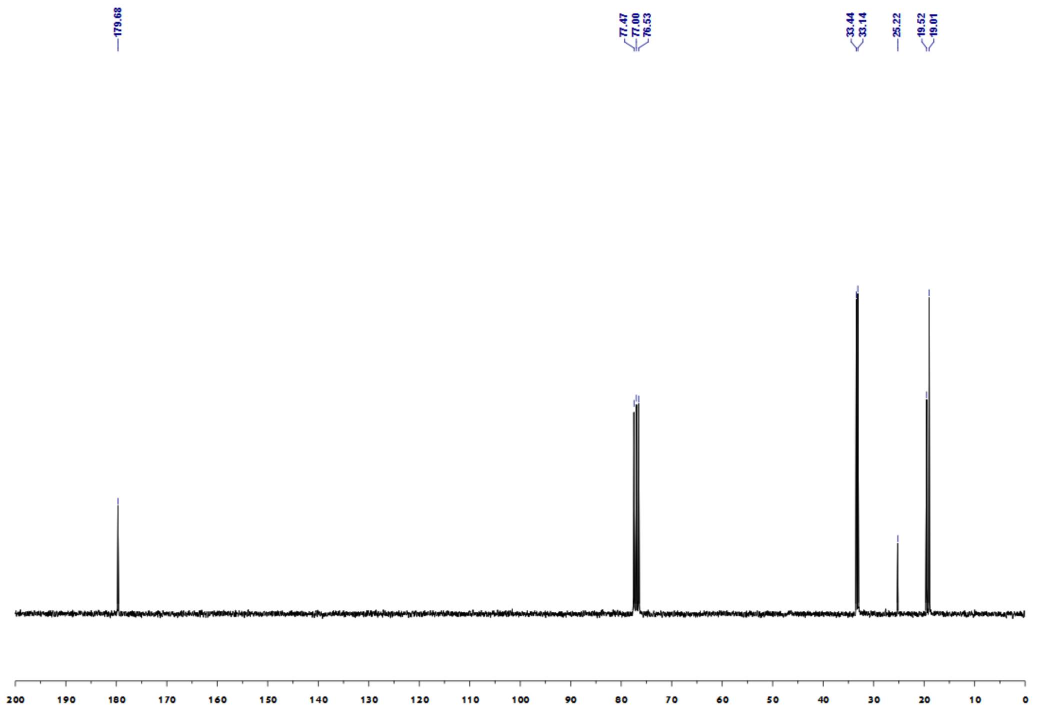

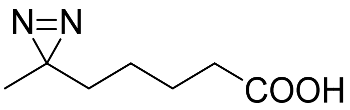
N=N
COOH

2c

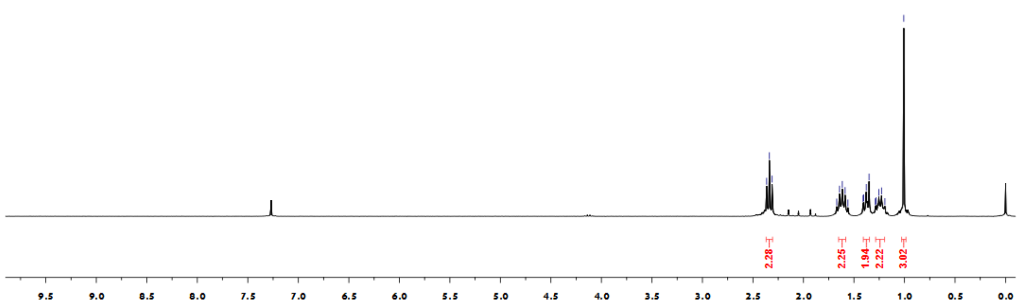
2.36
2.34
2.31
1.67
1.64
1.62
1.59
1.56
1.41
1.40
1.38
1.35
1.29
1.28
1.25
1.23
1.19
1.01
2.28
2.25
1.94
2.22
3.02

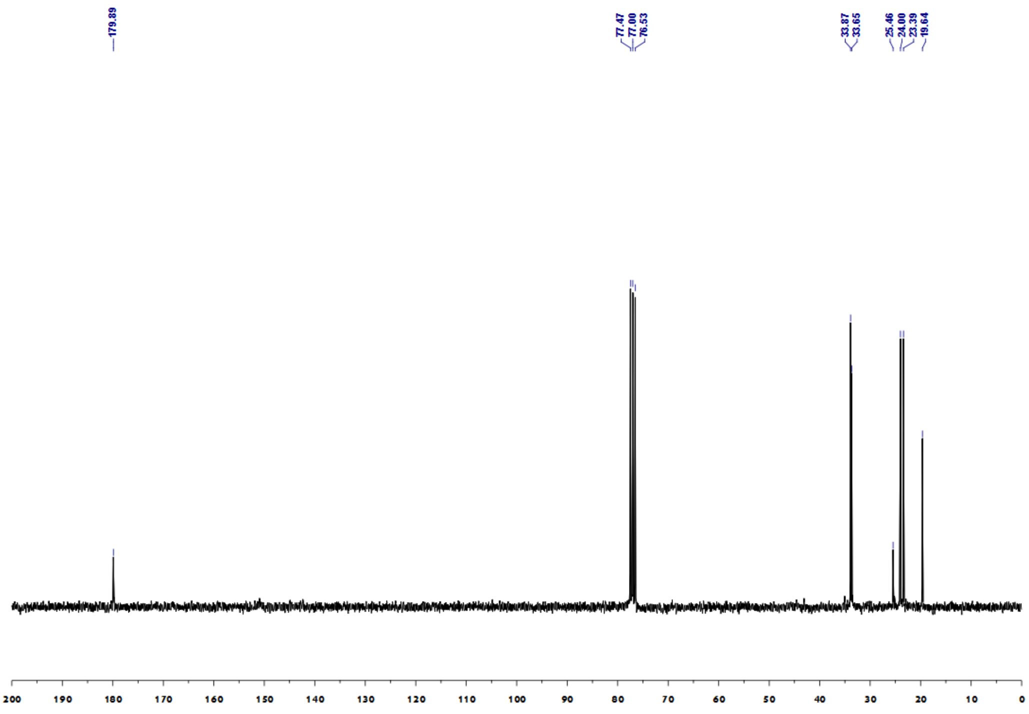
179.89
77.47
77.00
76.53
33.87
33.65
25.46
24.00
23.39
19.64

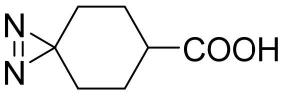
N
N
COOH

2d

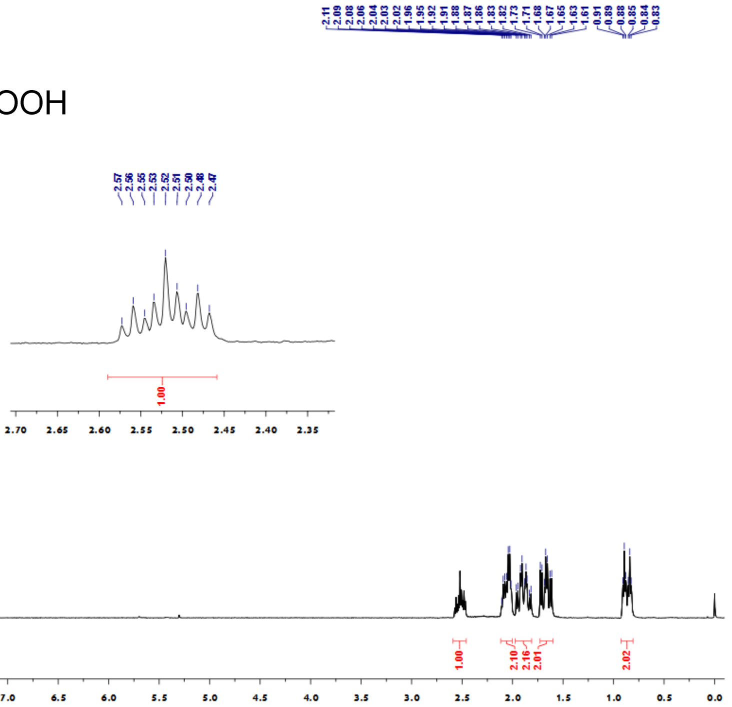
2.11 2.09 2.08 2.06 2.04 2.03 2.02 1.96 1.95 1.92 1.91 1.88 1.87 1.86 1.83 1.82 1.73 1.71 1.68 1.67 1.65 1.63 1.61 0.91 0.89 0.88 0.85 0.84 0.83
2.57 2.56 2.55 2.53 2.52 2.51 2.50 2.48 2.47
1.00
2.70 2.65 2.60 2.55 2.50 2.45 2.40 2.35
1.00 2.10 2.16 2.01 2.02
9.5 9.0 8.5 8.0 7.5 7.0 6.5 6.0 5.5 5.0 4.5 4.0 3.5 3.0 2.5 2.0 1.5 1.0 0.5 0.0

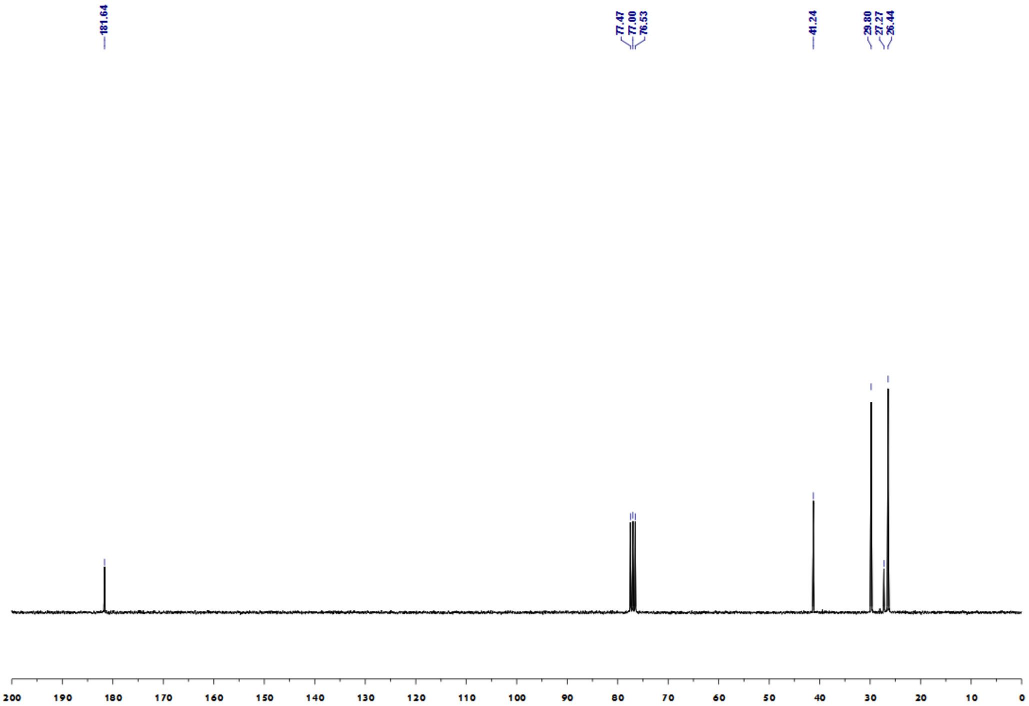
181.64
77.47 77.00 76.53
41.24
29.80 27.27 26.44
200 190 180 170 160 150 140 130 120 110 100 90 80 70 60 50 40 30 20 10 0

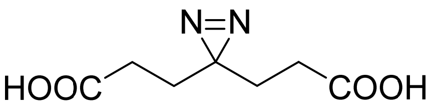
N=N
HOOC
COOH

2e

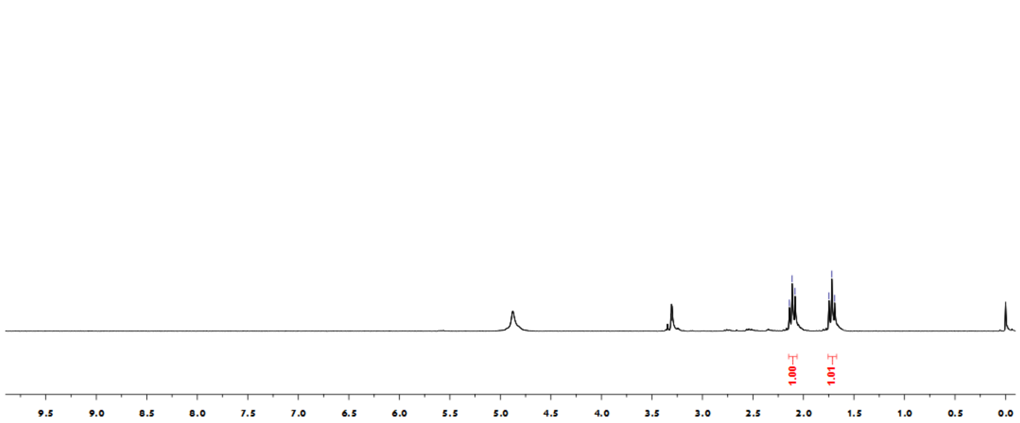
2.14
2.11
2.08
1.75
1.72
1.69
1.00
1.01
9.5 9.0 8.5 8.0 7.5 7.0 6.5 6.0 5.5 5.0 4.5 4.0 3.5 3.0 2.5 2.0 1.5 1.0 0.5 0.0

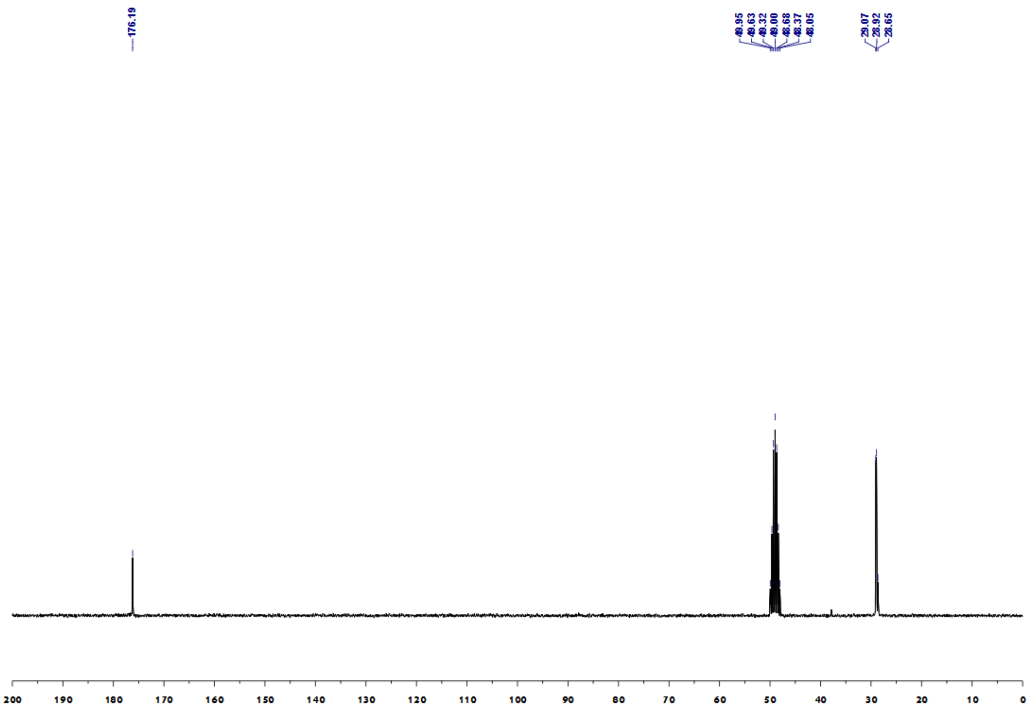
176.19
49.95
49.63
49.32
49.00
48.68
48.37
48.05
29.07
28.92
28.65
200 190 180 170 160 150 140 130 120 110 100 90 80 70 60 50 40 30 20 10 0

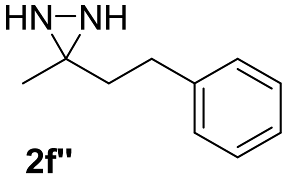
HN–NH

2f''

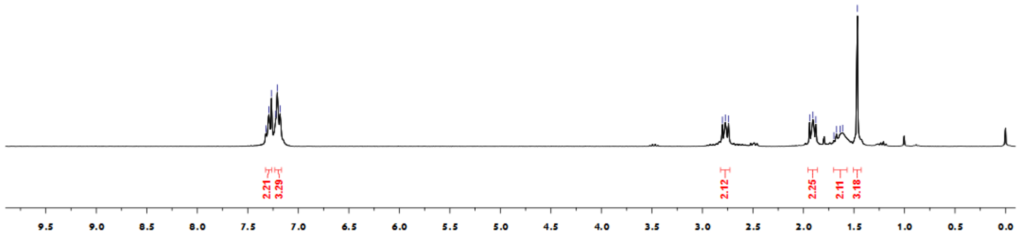
7.32
7.29
7.27
7.22
7.21
7.18
2.80
2.77
2.74
1.94
1.91
1.88
1.70
1.67
1.64
1.61
1.47
2.21
3.29
2.12
2.25
2.11
3.18
9.5 9.0 8.5 8.0 7.5 7.0 6.5 6.0 5.5 5.0 4.5 4.0 3.5 3.0 2.5 2.0 1.5 1.0 0.5 0.0

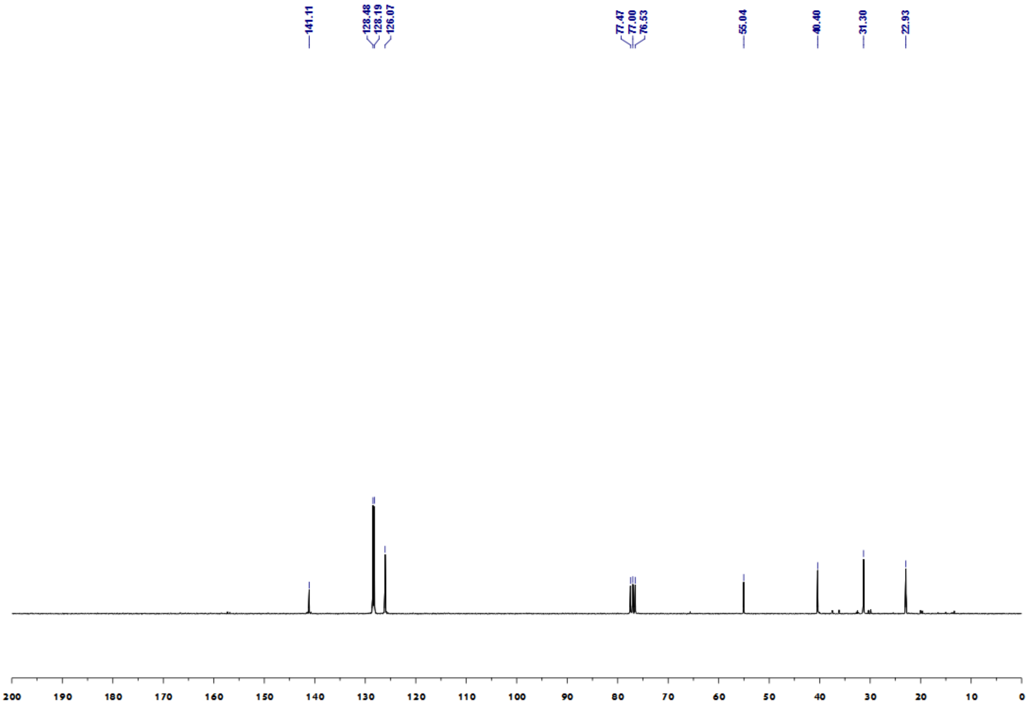
141.11
128.48
128.19
126.07
77.47
77.00
76.53
55.04
40.40
31.30
22.93
200 190 180 170 160 150 140 130 120 110 100 90 80 70 60 50 40 30 20 10 0

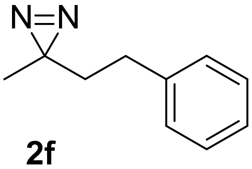
N=N

2f

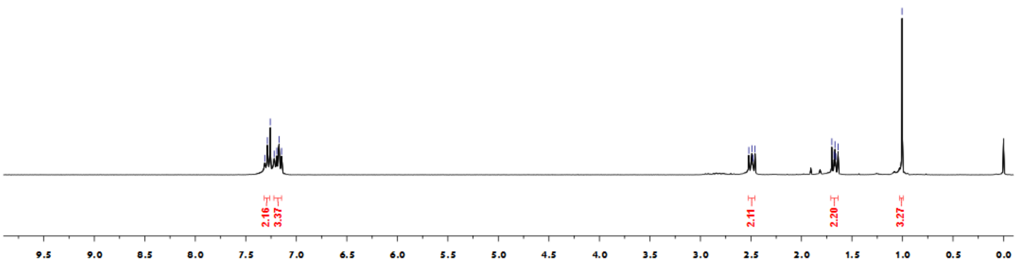
7.31
7.29
7.26
7.22
7.19
7.17
7.15
2.52
2.49
2.48
2.46
1.70
1.68
1.67
1.66
1.64
1.00
2.16
3.37
2.11
2.20
3.27
9.5
9.0
8.5
8.0
7.5
7.0
6.5
6.0
5.5
5.0
4.5
4.0
3.5
3.0
2.5
2.0
1.5
1.0
0.5
0.0

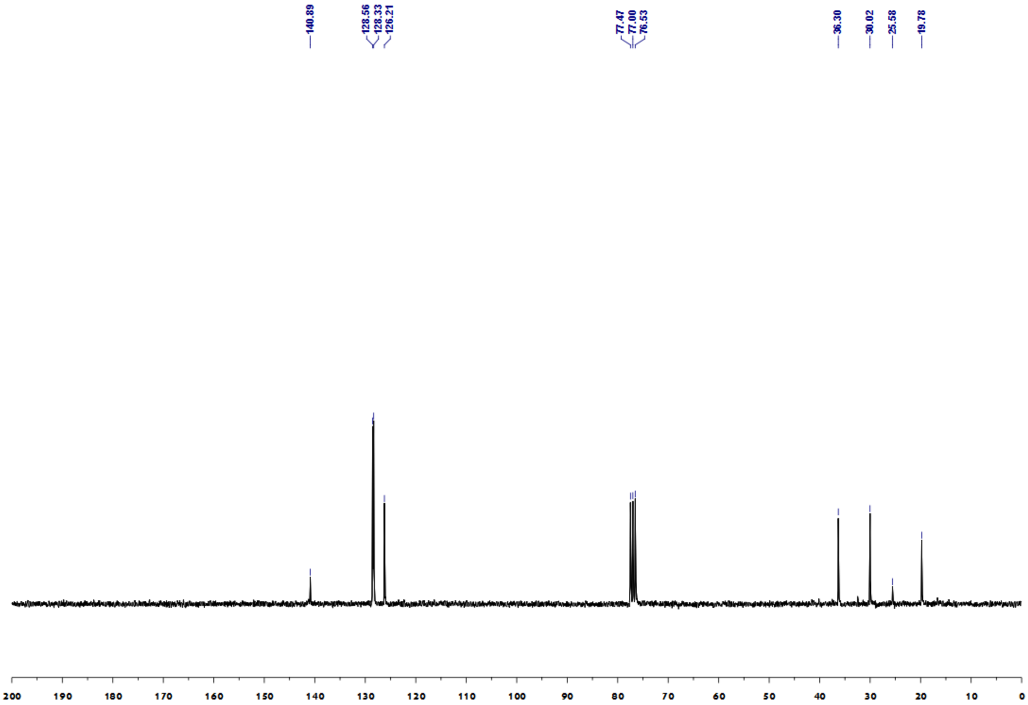
140.89
128.56
128.33
126.21
77.47
77.00
76.53
36.30
30.02
25.58
19.78
200
190
180
170
160
150
140
130
120
110
100
90
80
70
60
50
40
30
20
10
0

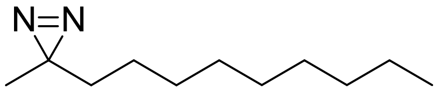
N=N

2g

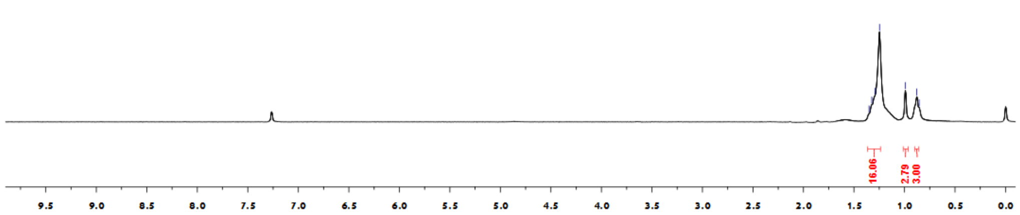
1.35
1.32
1.29
1.25
0.99
0.88
0.86
16.06
2.79
3.00
9.5 9.0 8.5 8.0 7.5 7.0 6.5 6.0 5.5 5.0 4.5 4.0 3.5 3.0 2.5 2.0 1.5 1.0 0.5 0.0

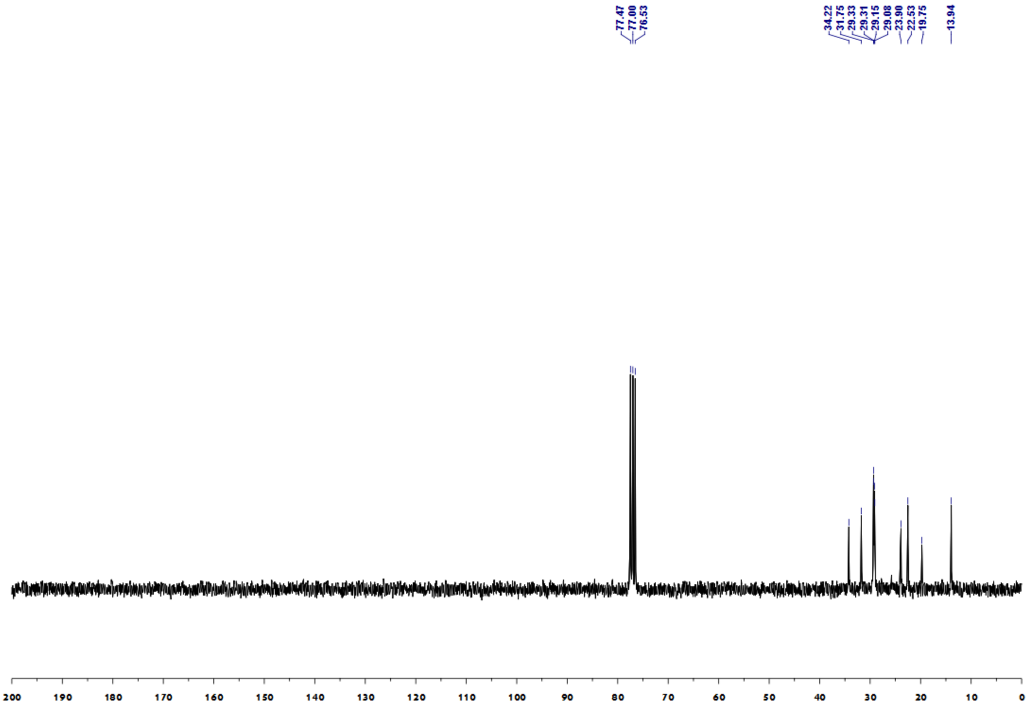
77.47
77.00
76.53
34.22
31.75
29.33
29.31
29.15
29.08
23.90
22.53
19.75
13.94
200 190 180 170 160 150 140 130 120 110 100 90 80 70 60 50 40 30 20 10 0

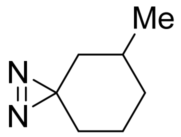

2h

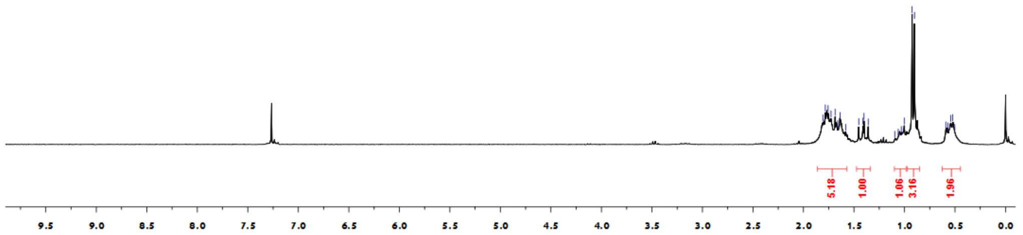

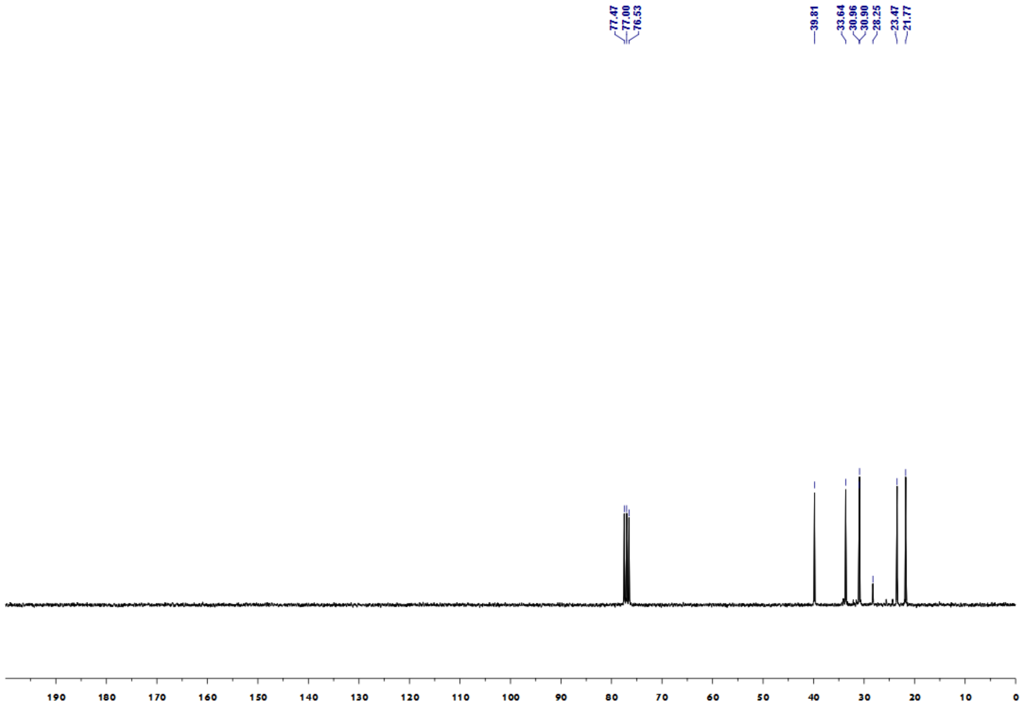

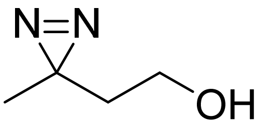
N=N
OH

2i

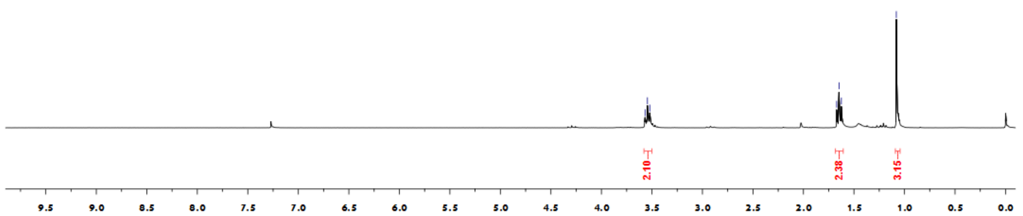
3.57
3.54
3.52
1.67
1.65
1.62
1.08
2.10
2.38
3.15
9.5 9.0 8.5 8.0 7.5 7.0 6.5 6.0 5.5 5.0 4.5 4.0 3.5 3.0 2.5 2.0 1.5 1.0 0.5 0.0

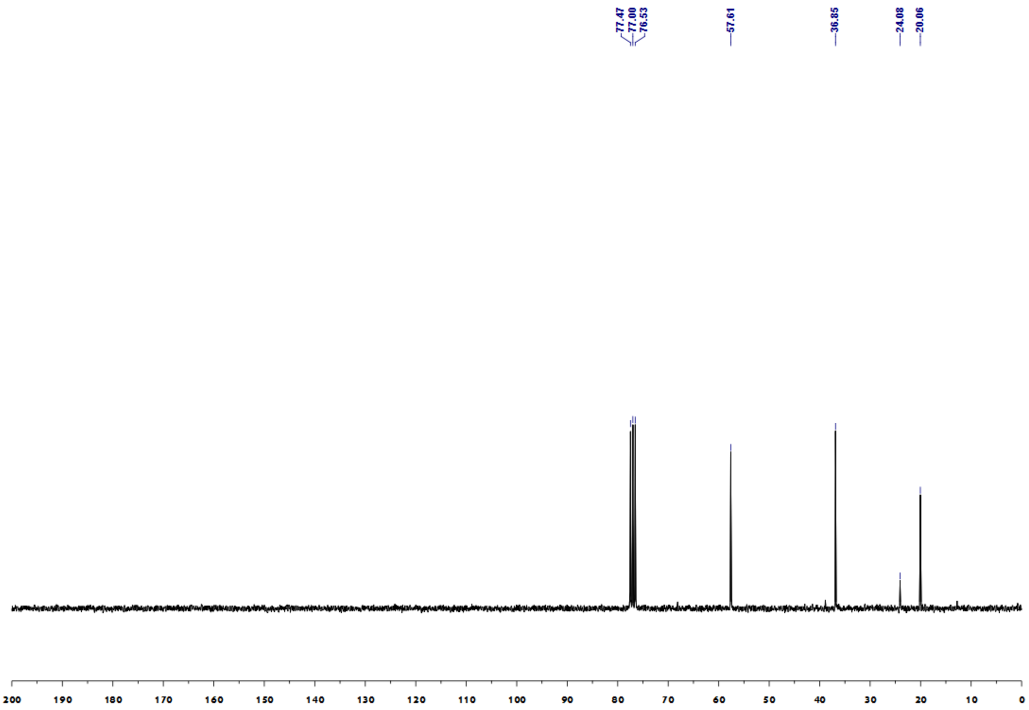
77.47
77.00
76.53
57.61
36.85
24.08
20.06
200 190 180 170 160 150 140 130 120 110 100 90 80 70 60 50 40 30 20 10 0

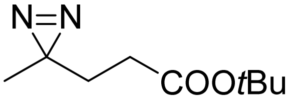
N=N
COOtBu

2j

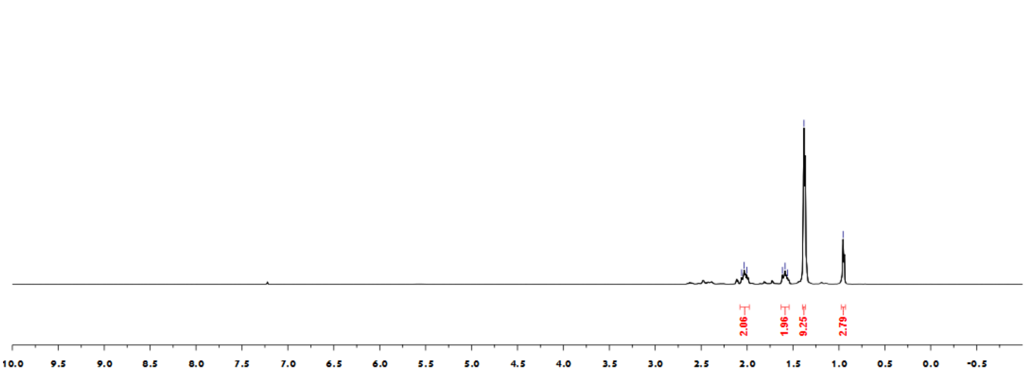
2.06
2.03
2.00
1.62
1.59
1.56
1.38
0.95
2.06
1.96
9.25
2.79
10.0
9.5
9.0
8.5
8.0
7.5
7.0
6.5
6.0
5.5
5.0
4.5
4.0
3.5
3.0
2.5
2.0
1.5
1.0
0.5
0.0
-0.5

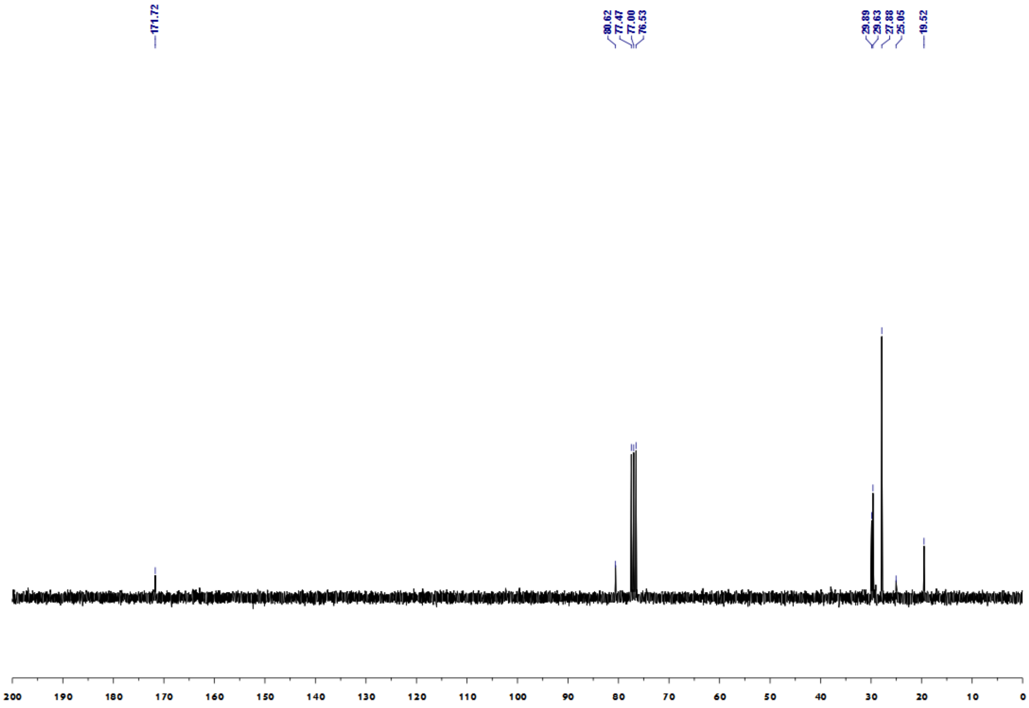
171.72
80.62
77.47
77.00
76.53
29.89
29.63
27.88
25.05
19.52
200
190
180
170
160
150
140
130
120
110
100
90
80
70
60
50
40
30
20
10
0
